# Development of a Novel Method for Analyzing *Pseudomonas aeruginosa* Twitching Motility and Its Application to Define the AmrZ Regulon

**DOI:** 10.1371/journal.pone.0136426

**Published:** 2015-08-26

**Authors:** Binjie Xu, Daniel J. Wozniak

**Affiliations:** 1 Department of Microbiology, The Ohio State University, Columbus, Ohio, 43210, United States of America; 2 Center for Microbial Interface Biology, The Ohio State University, Columbus, Ohio, 43210, United States of America; 3 Department of Microbial Infection and Immunity, The Ohio State University, Columbus, Ohio, 43210, United States of America; The Scripps Research Institute and Sorrento Therapeutics, Inc., UNITED STATES

## Abstract

Twitching motility is an important migration mechanism for the Gram-negative bacterium *Pseudomonas aeruginosa*. In the commonly used subsurface twitching assay, the sub-population of *P*. *aeruginosa* with active twitching motility is difficult to harvest for high-throughput studies. Here we describe the development of a novel method that allows efficient isolation of bacterial sub-populations conducting highly active twitching motility. The transcription factor AmrZ regulates multiple *P*. *aeruginosa* virulence factors including twitching motility, yet the mechanism of this activation remains unclear. We therefore set out to understand this mechanism by defining the AmrZ regulon using DNA microarrays in combination with the newly developed twitching motility method. We discovered 112 genes in the AmrZ regulon and many encode virulence factors. One gene of interest and the subsequent focus was *lecB*, which encodes a fucose-binding lectin. DNA binding assays revealed that AmrZ activates *lecB* transcription by directly binding to its promoter. The *lecB* gene was previously shown to be required for twitching motility in *P*. *aeruginosa* strain PAK; however, our *lecB* deletion had no effect on twitching motility in strain PAO1. Collectively, in this study a novel condition was developed for quantitative studies of twitching motility, under which the AmrZ regulon was defined.

## Introduction

Microorganisms are ubiquitous in all types of habitats, ranging from hot springs in deep seas to underneath thick ice sheets in the Antarctic continent. Occasionally, environmental bacteria such as *Pseudomonas aeruginosa* find a human host and become pathogenic. Efficient colonization and dissemination of *P*. *aeruginosa* is dependent on its multiple motility mechanisms–swimming, twitching, and swarming [1–4]. Under laboratory settings, swimming motility occurs in liquid culture or on semi-solid surfaces (0.3% agar) yet this is suppressed on solid surfaces [1,5]. In contrast, swarming motility is observed on softer agar surfaces (0.5–0.7% agar) compared to twitching motility (TM) (1% agar) [2,4]. Therefore, growth conditions significantly impact activities of different motility organelles. TM is conducted by type IV pili (TFP), which also contribute to adherence onto epithelial cells, biofilm formation, DNA uptake, and virulence [6–12].

TFP are present in a variety of bacterial genera including Proteobacteria, Cyanobacteria and Firmicutes [13]. The TFP machinery mediates cell motion by repeating cycles of pili extension, tethering to surfaces and retraction. Almost 40 *P*. *aeruginosa* genes have been shown to be involved in TM [14], and assembly of the TFP apparatus is efficiently coordinated by these gene products. Briefly, in *P*. *aeruginosa*, pre-major pilin PilA and other minor pilins are processed by the peptidase PilD, assembled into the pilus fiber by the ATPase PilB, and translocated across the cytoplasmic membrane via the transmembrane protein PilC. With the guidance by the alignment complex composed of PilMNOP and potentially FimV, polymerized pilus filaments pass through the outer membrane complex (PilQ and PilF) [12]. During retraction, pilus filaments are depolymerized by the ATPase PilT with probable assistance from PilU [12,13].

TM has been studied by multiple approaches such as macroscopic observations of colony edges, macro-/micro-scopic subsurface twitching assays and video-microscopy in flow cells [15–18]. Macroscopic observations of colony edges provided the platform for two independent transposon mutant screening studies [19,20]. Currently the most popular method is the macroscopic subsurface twitching assay, in which bacteria are stabbed underneath the agar and grow at the agar/Petri dish interface [14–16]. At interstitial surfaces, TM-proficient *P*. *aeruginosa* produces both TM-active and TM-inactive sub-populations [15]. Despite its convenience for imaging studies, this method is limited by the difficulty to recover sufficient quantities of TM-active bacteria from underneath the agar. This might explain why limited high-throughput (a.k.a. omics) studies have been published focusing specifically on TM-active sub-populations. We therefore designed a novel approach to achieve this goal. In this method, cellophane sheets placed above the agar surface allow diffusion of nutrients from the underlying media, supporting bacterial growth; more importantly, cellophane sheets provide moist and smooth surfaces required for TM activation. We showed that TM-proficient *P*. *aeruginosa* strains exhibited active TM under this condition and were easily distinguishable from TM-deficient ones.

We next sought to take advantage of this approach to understand AmrZ-mediated regulation in *P*. *aeruginosa*. AmrZ (alginate and motility regulator Z) is a global regulator of multiple virulence factors, including the metabolism of bis-(3’,5’)-cyclic diguanylate (c-di-GMP), extracellular polysaccharide production, and flagella [21–24]. Although AmrZ was also shown to be necessary for TM, its mechanism requires further investigation [25]. Therefore, we harvested TM-active bacterial sub-populations with the cellophane-based approach, and used DNA microarrays to compare transcriptional profiles between WT *P*. *aeruginosa* and two isogenic *amrZ* mutants. The AmrZ regulon under this condition contained 112 genes, and the lectin-encoding gene *lecB* was the only one known necessary for TM [26] in this gene list. The fucose-specific LecB (formerly known as PA-IIL) is abundantly present intracellularly and on the outer membrane of *P*. *aeruginosa*, and it plays important roles in host cell adherence and reduction of the ciliary beat frequency of the airway epithelium [27–29]. We provide evidence that AmrZ activates *lecB* expression by specifically binding to its promoter. However, *lecB* overexpression was not sufficient to restore TM in the Δ*amrZ* mutant, indicating *lecB* is either not required for TM in strain PAO1 or AmrZ-dependent genes in addition to *lecB* are necessary for TM. In support of the former, we were unable to recapitulate the TM-deficient phenotype seen previously with the PAK Δ*lecB* mutant [26] in our PAO1 strain background.

In conclusion, in this study we developed a novel approach to harvest actively twitching *P*. *aeruginosa* cells, and applied this new method to define the AmrZ regulon.

## Materials & Methods

### Bacterial Strains, Plasmids, Oligonucleotides and Growth Conditions

Information of bacterial strains, plasmids, and oligonucleotides employed in this study is summarized in S1 Table. Bacteria (*Escherichia coli* and *Pseudomonas aeruginosa*) were grown as previously [30]. Briefly, *E*. *coli* strains were grown in LB (each liter LB contains 10 g tryptone, 5 g yeast extract, 5 g sodium chloride) or on LA (LB with 1.5% (w/v) agar). 100 μg/ml ampicillin was used to maintain plasmids in *E*. *coli*. All *P*. *aeruginosa* strains were grown in LBNS or on LANS (LB or LA without sodium chloride, respectively). In *P*. *aeruginosa*, 300 μg/ml carbenicillin was used to maintain plasmids. All strains were grown at 37°C unless stated otherwise. All oligonucleotides were ordered desalted from Sigma Aldrich.

In-frame deletion mutants were generated through overlap extension PCR using the gene replacement vector pEX18Ap [31,32]. Multi-copy pHERD20T-based plasmids were transferred into *P*. *aeruginosa* strains through bi-parental mating with the *E*. *coli* helper strain S17-1 [33,34].

### Subsurface Twitching Assays

Subsurface twitching assays were modified from a previously described study [14]. Briefly, a single colony of a strain of interest was resuspended in 400 μl LBNS, and P10 tips were immersed in the above suspension and used to stab through the agar of 1-day-old 1% LANS plates. Plates were incubated upright in a humid chamber at room temperature for 5 days, and imaged through white epiluminescence via a ChemicDoc imaging system (Bio-Rad).

### Twitching Motility Assays on Cellophane Sheets

Square sterile cellophane sheets (Bioexpress, catalog #: E-3077-14) were placed on top of 1-day-old 1% LANS plates. A single colony of each strain was resuspended in 400 μl LBNS, and 2 μl spotted in the middle of the sheet. After air-drying, plates were incubated upright in a humid chamber at room temperature for 48 h. Microscopic images were taken by the Olympus DP71 camera mounted onto the inverted light microscope Olympus CKX41.

### Quantitative Real-Time PCR (qRT-PCR)

To isolate total RNA, bacteria were harvested from different growth conditions. Cells grown on LANS plates were harvested by flooding with LBNS, while those on cellophane sheets were scraped off using P200 tips and then resuspended in LBNS. 1 ml bacteria (OD_600_ = 1) were lysed by the addition of 100 μl TE containing 1 mg/ml RNase-free lysozyme (Sigma Aldrich), and then underwent RNA isolation (RNeasy RNA isolation kit, Qiagen). To obtain cDNA, ~1 μg RNA was used for reverse transcription (Superscript III cDNA preparation kit, Invitrogen) following manufacturer protocols. Amounts of cDNA were determined by SYBR Green Mix (Bio-Rad) and CFX1000 thermal cyclers (Bio-Rad). The housekeeping gene *rpoD* was used as reference, and the ΔΔCt method was used to calculate relative gene expression [22,35].

### DNA Microarray Analysis

Bacteria from the “edge” sub-population on cellophane sheets were scraped off by P200 tips, and resuspended in fresh LBNS. Total RNA was isolated as for qRT-PCR. Thereafter, 10 μg RNA for each strain was used to generate cDNA libraries and then analyzed using GeneChip *P*. *aeruginosa* Genome Array (Affymetrix) following manufacture instructions. Microarray “.cel” raw files were analyzed with RMA normalization, BH adjust, cutoff of p.value = 0.05, lfc = 1, using “*affy*” and “*limma*” packages in Bioconductor, in the environment of R 3.0.2 [36–39]. Microarray raw and result files have been deposited in NCBI Gene Expression Omnibus (Accession number: GSE68066).

### DNA Binding Studies

Electrophoretic mobility shift assays (EMSA) were used to evaluate AmrZ-DNA binding as previously described [22]. Each EMSA reaction contained 5 nM [FAM]-labeled DNA. After electrophoresis, EMSA gels were imaged via a Typhoon scanner (GE Lifescience) with the following settings: fluorescence, PMT800, 520BP 40 CY2 Blue FAM, 100 μm pixel size.

## Results

### Development of a Novel TM-Promoting Condition for Easy Cell Harvest

**Fig 1:**
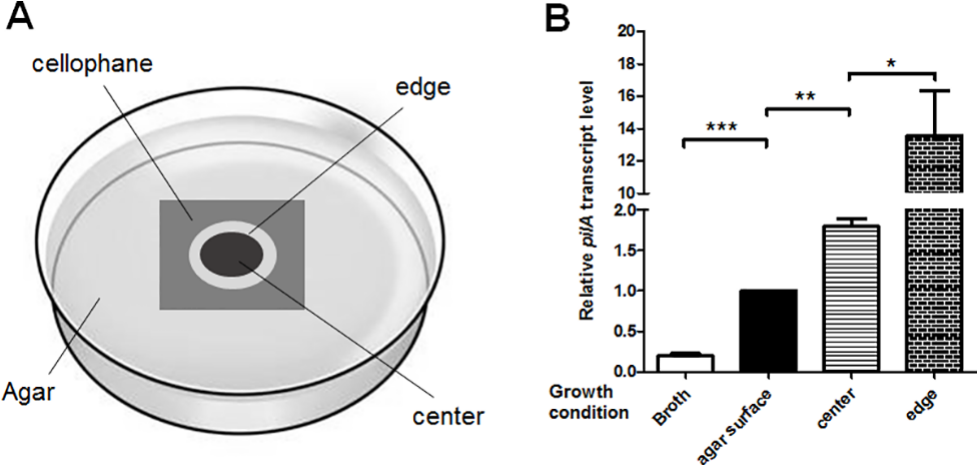
Cellophane-air interface stimulates *P*. *aeruginosa* twitching motility. (A.) Illustration of the cellophane-based TM-promoting condition. (B.) Quantification of *pilA* transcript levels from indicated growth conditions via qRT-PCR. “Broth” refers to overnight broth culture (stationary phase). Transcript levels of *pilA* were normalized to those isolated from single colonies on agar surfaces (“agar”) with *rpoD* as the reference gene. Unpaired two-tailed student t-test was used for statistical analysis of three independent experiments. *: P<0.05; **: P<0.01; ***: P<0.001.

Traditionally, TM activity is measured using subsurface twitching assays, in which bacteria grow at the agar-Petri dish interface [14,16]. One caveat of this method is the difficulty of harvesting sufficient actively twitching cells for downstream high-throughput analyses, such as transcriptomics and proteomics. Therefore, a novel growth condition using cellophane sheets was developed taking into consideration previous studies describing the significance of inert smooth surfaces (most important) and a humid environment [14,15]. It is also more convenient to harvest cells from the surface of cellophane sheets as opposed to the interstitial space between agar and Petri dishes. The design of this approach is shown in Fig 1A. Following 48-hour growth on cellophane, *P*. *aeruginosa* formed a large smear-like colony and we observed two distinct sub-populations of bacteria (“edge” and “center”; Fig 1A).

In order to evaluate if the cellophane-air interface promotes TM gene expression, we harvested WT *P*. *aeruginosa* (strain PAO1) from both sub-populations, and measured transcript levels of the type IV major pilin gene *pilA*. We used qRT-PCR to compare *pilA* mRNA levels among four different conditions: overnight broth culture (stationary phase), agar surfaces (heterogeneous populations), cellophane “edge”, and cellophane “center”. On agar surfaces, *pilA* expression was significantly higher compared to broth culture (Fig 1B), consistent with a previous study [40]. Strikingly, bacteria from the “edge” sub-population on cellophane sheets exhibited 14 or 8-fold elevation of the *pilA* mRNA level compared to cells harvested directly from agar surfaces or the “center” sub-population, respectively (Fig 1B). This observation is consistent with a previous finding using subsurface TM assays that the edge subpopulation produced significantly more PilA than the center [15]. Therefore, we conclude that under this newly developed condition, the “edge” sub-population on cellophane surfaces conducts highly active TM and is an ideal subject for downstream TM studies.

### TM Phenotypes of *P*. *aeruginosa* Strains on Cellophane Surfaces

**Fig 2:**
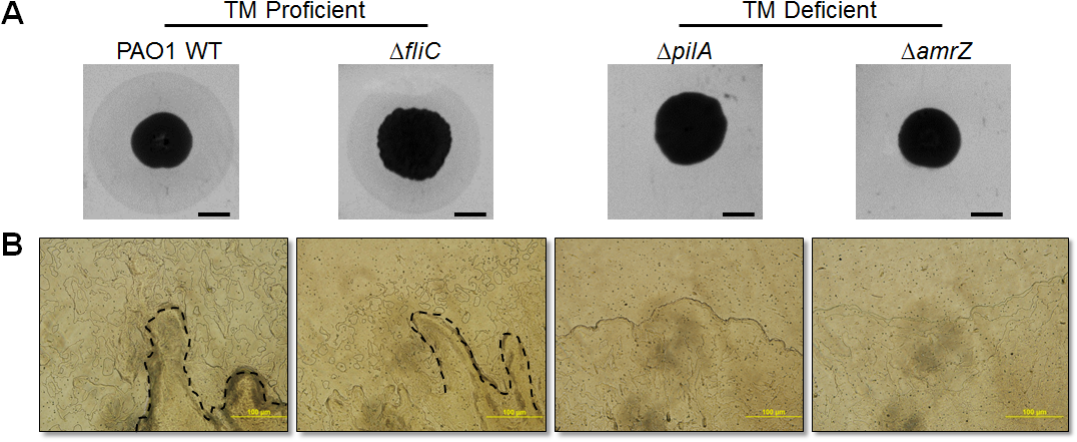
TM-proficient and TM-deficient strains retained their TM phenotypes on cellophane. (A.) TM measurement of four *P*. *aeruginosa* strains by subsurface twitching assays. Scale bar: 5 mm. (B.) Phase contrast microscopy of edge sub-populations of the same strains above cellophane sheets. Dashed lines illustrated tendril-like structures. Scale bar: 100 μm.

On surfaces where flagella-mediated swimming motility is suppressed, TFP provide opportunities for bacteria to explore new territories via TM (9, 34). The subsurface twitching assay is a valuable tool to distinguish TM-proficient from TM-deficient *P*. *aeruginosa*. We therefore sought to determine whether there is a TFP-dependent phenotype using the new cellophane-based method. Four *P*. *aeruginosa* strains were tested, including two TM-proficient strains (PAO1 and the flagella mutant Δ*fliC*) and two TM-deficient strains (Δ*pilA* and Δ*amrZ*). When the subsurface twitching method was used, TM-proficient strains exclusively formed twitching zones outside of the central colony as expected (Fig 2A). In cellophane-based assays, to discern differences between TM-proficient and TM-deficient strains, it was necessary to use phase contrast microscopy. These differences were not readily apparent by the naked eye. Microscopic examinations revealed any strain capable of TM (PAO1 and Δ*fliC*) displayed tendril-like structures at colony edges on cellophane sheets, which were absent in TM defective strains (Δ*pilA* and Δ*amrZ*) (Fig 2B). Notably, formation of these tendril-like structures is flagella-independent, as the flagella mutant Δ*fliC* still formed tendrils similar to its parental strain PAO1. In conclusion, this cellophane approach can serve as a new method to identify TM-proficient from deficient strains.

### Definition of the AmrZ Regulon under This TM-Promoting Condition

**Fig 3:**
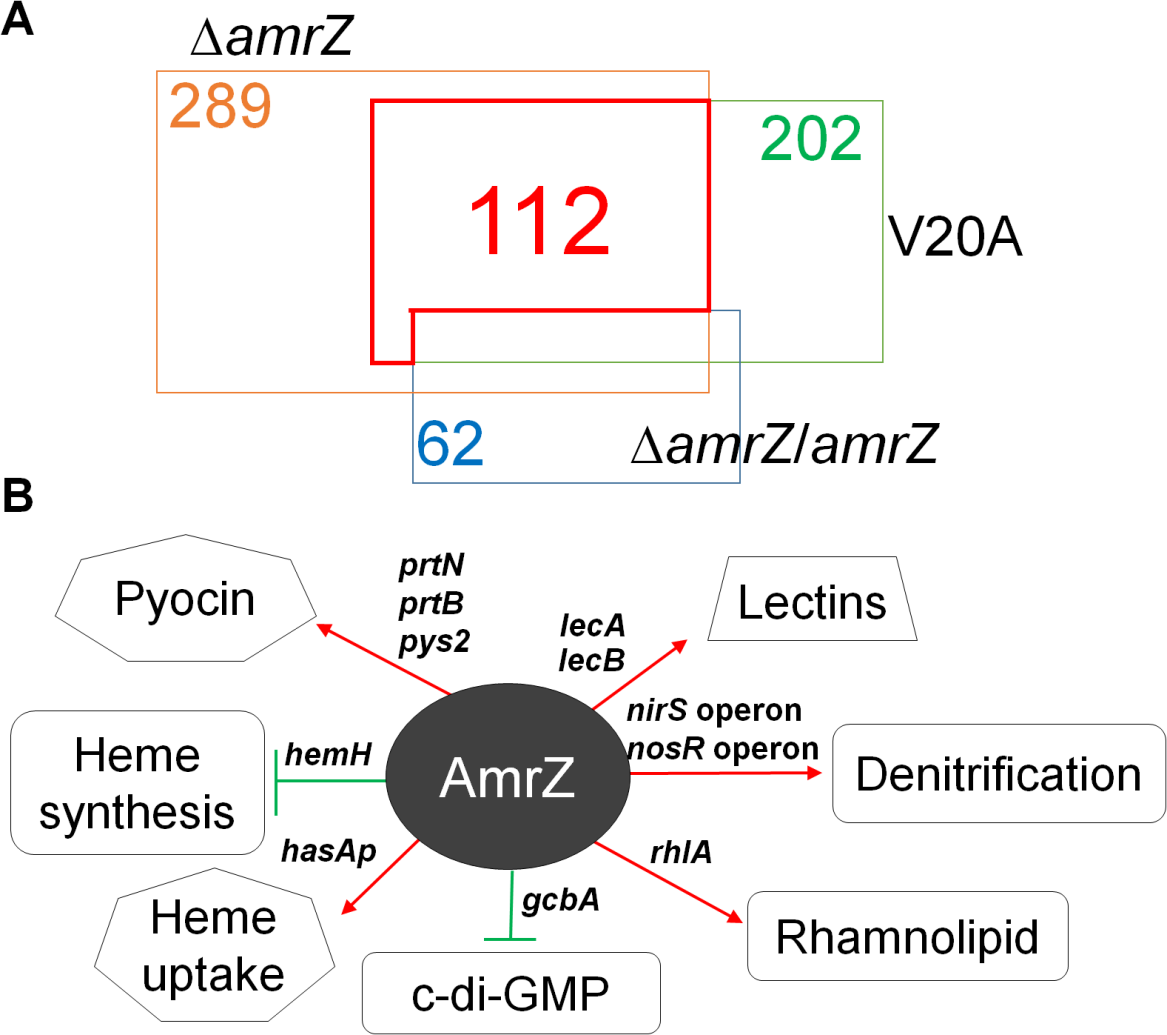
Identification of the AmrZ regulon from the TM-active “edge” sub-population. (A.) Venn diagram of the AmrZ regulon under the TM-promoting condition. 112 genes in the red box were included in the AmrZ regulon as they displayed differential expression between both *amrZ* mutants (Δ*amrZ* and AmrZV20A) and strains encoding WT AmrZ (PAO1 and the complemented strain Δ*amrZ*/*amrZ*). The other three numbers indicate the total number of genes that exhibited distinct expression from PAO1, respectively. (B.) Representative virulence pathways regulated by AmrZ. Red arrows indicate activation, while green bars represent repression.

It is unknown how the transcription factor AmrZ activates *P*. *aeruginosa* TM [25]. We hypothesized that AmrZ activates TM by modulating the transcription of target TM gene(s). Therefore, we sought to apply this newly-developed method to understand the mechanism of AmrZ-mediated TM activation. DNA microarray analyses were performed to identify AmrZ-dependent targets using cells harvested from “edge” sub-populations of the parental strain PAO1, two isogenic *amrZ* mutants (Δ*amrZ* and *AmrZV20A*), and a complemented Δ*amrZ* strain. AmrZV20A harbors a V20A point mutation that renders the protein deficient in DNA binding, a property required for its role in controlling TM [25,30]. The complemented Δ*amrZ* strain was constructed by placing the *amrZ* gene with its native promoter back into the Δ*amrZ* mutant at the native *amrZ* locus [25]. Our rationale was that any gene transcriptionally regulated by AmrZ would exhibit differential expression in *amrZ* mutants including Δ*amrZ* and AmrZV20A (AmrZ^-^) compared to strains expressing WT *amrZ* (PAO1 and the complemented strain; AmrZ^+^). It should be noted that during bacterial cell harvest, although *amrZ* mutants do not twitch and their colony edges appeared distinct from TM-proficient strains, we still harvested cells from their colony edge portions. As shown in Fig 3A, the AmrZ regulon contains 112 genes.

**Fig 4:**
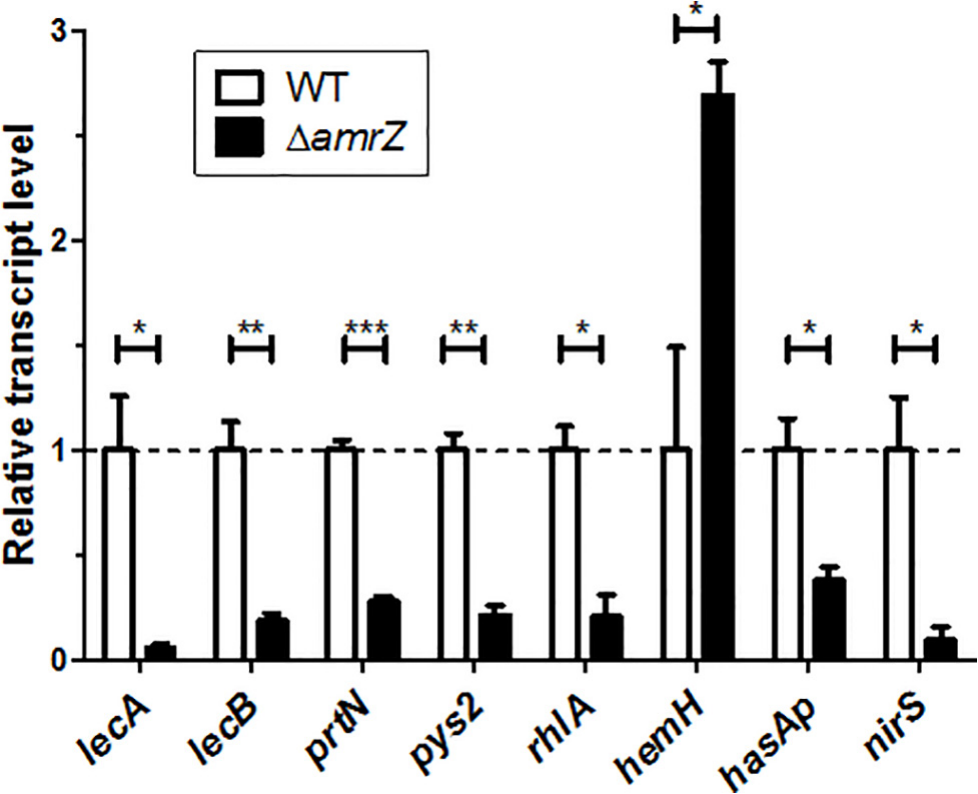
Verification of representative AmrZ-dependent genes. Bacteria growth conditions and RNA isolations were the same as for microarray analyses. Gene expression in the Δ*amrZ* mutant was compared to the parental strain PAO1 by qRT-PCR. The horizontal dashed line represented normalized mRNA levels as seen in the parental strain PAO1. Unpaired two-tailed student t-test was used for statistical analysis of three independent experiments. *: P<0.05; **: P<0.01; ***: P<0.001.

Multiple virulence pathways were affected by *amrZ* mutations (Fig 3B). A complete list of AmrZ-dependent genes is summarized in S2 Table. We confirmed mRNA levels of selected targets by qRT-PCR (Fig 4). Key virulence pathways regulated by AmrZ are categorized and discussed below.

- Diguanylate cyclase. AmrZ inhibited the expression of the diguanylate cyclase (DGC)-encoding gene *gcbA* (PA4843, also referred to as *adcA*), confirming previous findings [22,41]. GcbA is required for surface attachment and regulates flagella-mediated motility in *P*. *fluorescens*, and *gcbA* deletion led to reduced biofilms in *P*. *aeruginosa* [22,41]. Deletion of this DGC gene results in reduced c-di-GMP levels, which serves as a crucial second messenger regulating numerous processes involved in transitions between sessile and motile lifestyles [22,42,43].
- Lectins. Both *lecA* and *lecB* exhibited reduced expression in *amrZ* mutants. Galactose-specific lectin LecA and fucose-specific lectin LecB mediate *P*. *aeruginosa* adhesion and cytotoxicity towards epithelial cells, as well as contribute to lung damage in an acute mouse infection model [27,29]. In addition, *lecB* was shown to be necessary for robust TM and caseinolysis [26]. Since *lecB* was the only AmrZ-dependent gene found in our profiling studies known to be involved in TM, we focused on this in more detail (see below).
- Pyocin. AmrZ activated pyocin genes such as *pys2* and *prtN*. The *pys2* gene encodes the large component of the bacteriocin pyocin 2, and exhibits DNase activity [44]. PrtN functions as the activator for all types of pyocins [45].
- Rhamnolipids. AmrZ also activated *rhlA* expression. The *rhlA* gene encodes chain A of the rhamnosyltransferase, and is required for the synthesis of 3-(3-hy-droxyalkanoyloxy) alkanoic acids (HAAs) and the fatty acid moiety of rhamnolipids [46,47]. The biosurfactant rhamnolipids are involved in multiple processes such as swarming and sliding motilities, biofilm detachment, and have antimicrobial activities against other bacteria [2,3,48].
- Iron. Under this condition, AmrZ modulated iron metabolism via activating the expression of *hasAp* and repressing *hemH*. HasAp is necessary for haemoglobin uptake in *P*. *aeruginosa*, while the ferrochelatase HemH is the final enzyme involved in the heme biosynthesis pathway [49,50].
- Denitrification. AmrZ regulated transcription of multiple operons involved in denitrification. The majority of genes within *nirS* and *nosR* operons showed significantly reduced expression in *amrZ* mutants. These genes are required for anaerobic respiration using nitrate/nitrite as electron receptors, indicating the impact of AmrZ on denitrification.

### AmrZ Activates *lecB* Transcription by Directly Binding to Its Promoter

**Fig 5:**
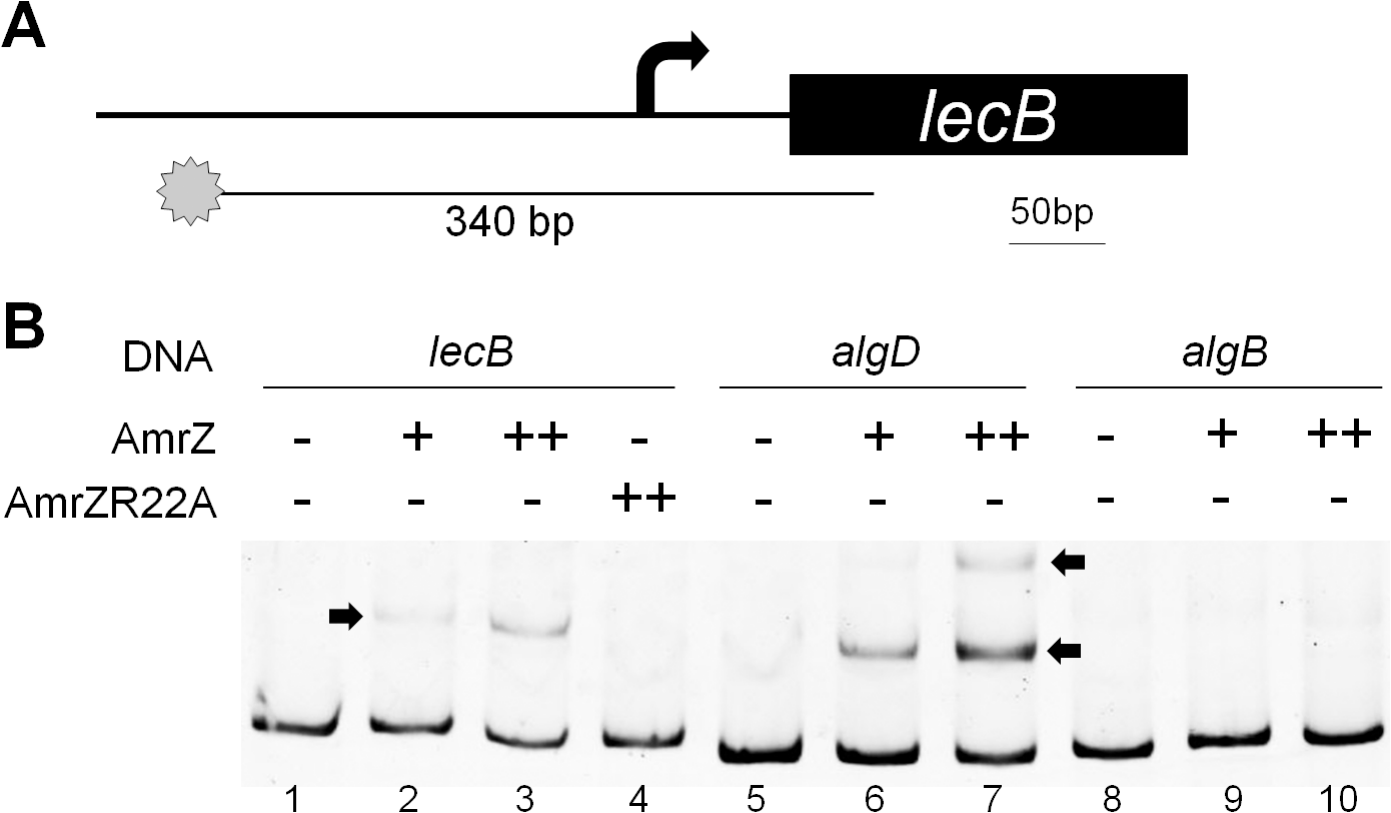
AmrZ specifically binds to the *lecB* promoter. (A.) Design of the *lecB* DNA fragment used in (B.). The star represents [6FAM] used to label the 5’ end of the *lecB* fragment. The arrow indicates the *lecB* transcription start site. (B.) EMSA was used to determine AmrZ binding to the *lecB* promoter, the positive control “*algD*” DNA, and the negative control “*algB*” DNA. Black arrows point at delayed mobility of DNA. Two AmrZ variants (AmrZ and the DNA binding-deficient variant AmrZR22A) were used in the experiment. “-“: No protein added; “+”: 50 nM protein; “++”: 100nM protein.

One goal in this study was to use this novel approach to identify AmrZ-dependent genes necessary for TM. As discussed above, only *lecB* within the defined AmrZ regulon was reported to be necessary for TM [26]. Therefore, we determined if AmrZ activates *lecB* transcription directly by binding to its promoter. A 5’-[6FAM]-labeled DNA fragment containing the *lecB* promoter region was amplified (Fig 5A), and its binding by AmrZ was determined through the electrophoretic mobility shift assay (EMSA). Addition of AmrZ caused mobility retardation of this DNA fragment, suggesting AmrZ-DNA binding. Moreover, this retardation required a DNA binding-proficient AmrZ, since the DNA binding deficient protein AmrZR22A was unable to cause a shift. In this EMSA assay, promoter fragments from *algD* and *algB* were used as positive and negative controls respectively. From this, we conclude that AmrZ specifically binds to the *lecB* promoter.

### The *lecB* Gene Is Not Necessary for TM in *P*. *aeruginosa* PAO1

**Fig 6:**
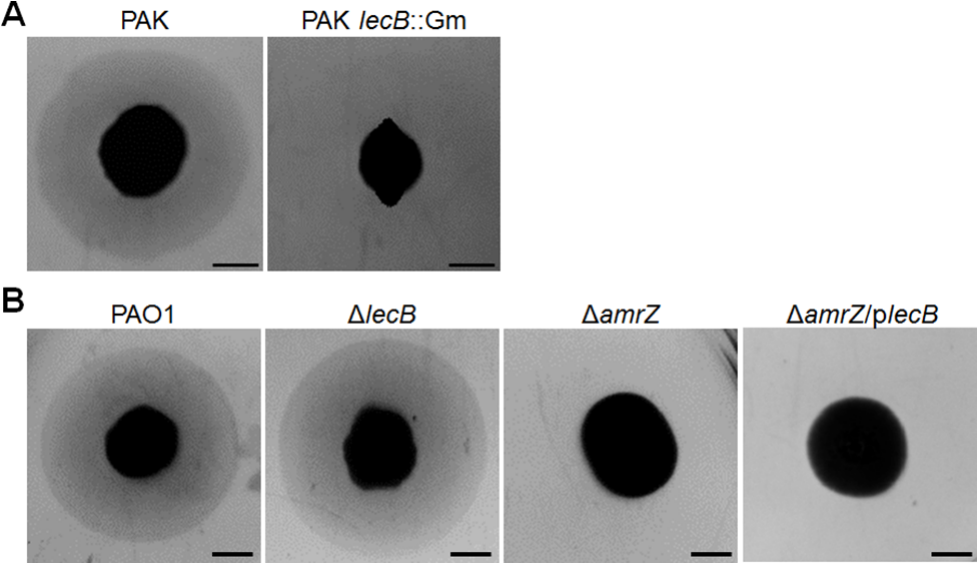
The *lecB* gene is required for TM in *P*. *aeruginosa* strain PAK, but not in strain PAO1. (A.) TM of the parental strain PAK and its derivative *lecB*::Gm^R^ via subsurface twitching assays [26]. (B.) Subsurface twitching assays of strains in PAO1 background. In the Δ*amrZ* strain with the p*lecB* plasmid, 1% arabinose was added as the inducer. Scale bar: 5 mm.

We hypothesized that since *lecB* was the only AmrZ-dependent gene known to be required for TM, *lecB* ectopic expression in the Δ*amrZ* mutant would restore TM. Therefore, we placed the *lecB* gene into the backbone of the arabinose-inducible vector pHERD20T. However, pHERD20T-*lecB* failed to restore TM in the Δ*amrZ* mutant after arabinose induction (Fig 6B).

In *P*. *aeruginosa* strain PAK, *lecB* deletion led to the absence of surface pili and defective TM (Fig 6A) [26]. Therefore, to examine the role of *lecB* in the PAO1 strain, we constructed an in-frame Δ*lecB* mutant, and measured its TM via the subsurface twitching assay. Surprisingly, the Δ*lecB* mutant displayed normal TM compared to its parental strain PAO1 (Fig 6B). This result was confirmed by microscopic observations using the cellophane-based method (S1 Fig).

## Discussion

In this study, we developed a novel cellophane-based approach for harvesting TM-active bacterial cells. This approach provides the convenience of recovering enough cells for downstream studies, the validity of which was confirmed by multiple assays. We then applied this method to define the AmrZ regulon through DNA microarray analyses and discover AmrZ-dependent genes. Absence of functional AmrZ led to global changes in gene expression, including the lectin-encoding gene *lecB*. We also revealed that *lecB* was not required for TM in *P*. *aeruginosa* strain PAO1.

*P*. *aeruginosa* is capable of thriving under diverse environments. Adaptation into new environments usually requires detection of external stimuli, intracellular signal transduction, and corresponding responses. In this study, we showed that *pilA* transcript levels were 69 times higher when grown on cellophane sheets than in broth culture. This suggests that *P*. *aeruginosa* has a remarkable capability of maintaining low *pilA* expression in liquid culture, but activating it when grown on surfaces, especially smooth ones such as cellophane sheets. Presumably minimal *pilA* expression in broth culture may conserve resources for bacteria when TFP are not necessary. In contrast, on surfaces where TFP are critical for migration, *pilA* expression is enhanced. On cellophane sheets, cells appeared to differentiate into “edge” and “center” sub-populations, and the “edge” sub-population displayed significantly higher TM activities than “center”, similar to previous findings using the subsurface twitching assay [15]. Activation of *P*. *aeruginosa* TFP responses on surfaces involves effective surface recognition, which requires multiple systems, including but likely not limited to the Wsp signal transduction system, the Chp chemotaxis system, and PilY1 in the TFP system [43,51–54]. Surface attachment resulted in phosphorylation and clustering of the response regulator WspR and an increased level of the second messenger cAMP, leading to a rise of the c-di-GMP concentration and the activation of the cAMP-binding regulator Vfr, respectively [43,54]. Downstream effects involve elevated PilY1 expression and its secretion by the TFP system, serving as one plausible mechanism to explain our observation of increased *pilA* expression on surfaces versus liquid.

Twitching motility is a well-coordinated group behavior, resulting in the formation of “bacterial rafts” on interstitial spaces [15,55]. Our microscopic observations on cellophane sheets were consistent with those findings. Tendril-like structures were observed at leading edges of exclusively TM^+^ *P*. *aeruginosa* similar to those rafts described previously. It is intriguing how bacteria aggregate and properly arrange themselves in these rafts. The Psl exopolysaccharide fibers and extracellular DNA (eDNA) are two factors involved in this phenomenon. Previous studies observed that Psl fibers held bacteria together and linked aggregates, which were absent in TM-deficient strains [56,57]. In another elegant study, Gloag *et al*. showed that DNase I treatment inhibited the formation of lattice-like networks formed by TFP [55]. Potential roles of Psl and DNA on TM under our cellophane condition require further investigation.

With this new TM-promoting approach, we defined the AmrZ regulon through DNA microarray analyses. Absence of functional AmrZ led to global gene expression changes, and multiple pathways associated with pathogenesis were impacted. Previously we and others defined the AmrZ regulon via RNA-Seq of mid-log phase bacteria grown in liquid culture, one in *P*. *aeruginosa* and the other one in *P*. *fluorescens* [22,58]. A comparison was made between the current DNA microarray analysis and the previous *P*. *aeruginosa* RNA-seq study [22]. The AmrZ regulons from both studies shared multiple genes, such as the DGC-encoding gene *gcbA*, and hypothetical genes PA0102, PA3235, etc. (S2 Table). However, there were significant differences between these two studies. First, growth conditions were dissimilar in these two studies. We and others have shown that *pilA* expression varies depending on growth conditions (activated on surfaces while repressed in liquid) (Fig 1B; [40]), and it is likely that other genes would behave differently in these conditions. Second, in the RNA-Seq study, the comparison was made between a Δ*amrZ* mutant and an *amrZ*-overexpressing strain, which mimics alginate-overproducing mucoid *P*. *aeruginosa* [22]. However, in the current study all strains are in the non-mucoid PAO1 background and have significantly lower AmrZ levels, which may lead to distinct regulatory outcomes.

Our DNA microarray results compared transcriptional profiles between AmrZ^-^ and AmrZ^+^ strains. Within the four strains used, one AmrZ^-^ strain encodes a DNA binding-deficient variant AmrZV20A and is TM deficient, while the complemented strain re-obtained WT AmrZ and has restored TM [25]. Thus, the comparison between AmrZ^-^ and AmrZ^+^ strains should only reveal targets that are modulated by DNA binding-proficient AmrZ. It was therefore surprising that 62 genes had differential expression between the complemented strain and PAO1. However, the majority of these genes had differential expression only slightly above the 2-fold threshold. Only 10 genes showed more than 3-fold difference, and the largest difference was 6.4-fold. Nevertheless, the possibility of unknown artifacts introduced during strain construction cannot be excluded. Therefore, these 62 genes have been excluded from the AmrZ regulon.

This study discovered that both lectin-encoding genes *lecA* and *lecB* exhibited differential expression between AmrZ^-^ and AmrZ^+^ strains. In addition, AmrZ specifically bound to the *lecB* promoter, indicating direct AmrZ activation. Interestingly, in a previous study when ChIP-Seq was used to determine AmrZ binding sites throughout the *P*. *aeruginosa* genome, AmrZ was not observed to bind to the *lecB* promoter [22]. This could also be attributed to different growth conditions. It is plausible that in broth culture and possibly other conditions, certain signals suppress AmrZ binding to the *lecB* promoter thereby preventing its transcriptional activation.

Our original hypothesis was that AmrZ activates TM through *lecB*, which was shown to be required for TM in *P*. *aeruginosa* strain PAK. Surprisingly, our Δ*lecB* mutant in PAO1 did not show a TM defect, in contrast to the study in the PAK strain background [26]. To determine if the *lecB* gene differs between these two strains, we sequenced the *lecB* coding and flanking sequences. There was a single nucleotide variation at the 48^th^ nucleotide in the *lecB* coding sequence: a deoxythymidine in PAO1 and a deoxycytidine in PAK (data not shown). However, this polymorphism does not lead to a change in the amino acid sequence, since both codons (ACU and ACC) encode threonine. Nevertheless, it is possible that with a different genetic background the *lecB* gene in PAK might play a different role compared to our PAO1 strain. A comprehensive transposon mutant library was constructed in another PAO1 strain MPAO1 and screened to identify TM-necessary genes [20]. This screening did not identify any *lecB* mutant that lost TM. Meanwhile, there was no overlap between *tfp* genes identified in the transposon mutant screening [20] and AmrZ-dependent genes in the current study. Therefore, the AmrZ regulon described in this study does not contain any known gene(s) necessary for TM. We also considered the possibility that AmrZ regulates TM indirectly by modulating c-di-GMP levels since this signaling molecule affects TM [59,60]. We reasoned that AmrZ represses the DGC gene *gcbA*, and loss of functional AmrZ leads to increased c-di-GMP levels, which could indirectly inhibit TM. To test this, a plasmid carrying a gene encoding the phosphodiesterase PA2133 was introduced into the Δ*amrZ* mutant. This has been used in the past to reduce c-di-GMP levels in *P*. *aeruginosa* [61]. However, PA2133 overexpression did not restore TM in Δ*amrZ* (S2 Fig). Therefore, despite our efforts, the mechanism for AmrZ-mediated control of TM therefore remains unknown. It is a formal possibility that AmrZ may regulate TM at a post-transcriptional level, e.g. via small RNAs, during translation or through protein-protein interactions. This is less likely because of the stringent requirement of DNA binding-proficient AmrZ for TM. The most likely explanation therefore is that TM genes controlled by AmrZ have not been identified in previous genetic strategies to identify TM-defective strains. Our current aim is to use alternative approaches to identify these targets.

Overall, this study described a novel TM-promoting approach that facilitates the harvest of TM-active cells for downstream transcriptomics studies. We defined the AmrZ regulon under this growth condition, and uncovered the role of AmrZ as a global regulator in *P*. *aeruginosa*. This novel approach may be applied to other bacterial species capable of TM and facilitate more high-throughput research aiming at understanding regulatory mechanisms of this unique form of motility.

## Supporting Information

S1 Fig *lecB* is required for TM in PAK, but not in PAO1 using the cellophane-based method. Phase contrast microscopy was used to examine edge subpopulations of PAO1, PAK, and their respective *lecB* mutants after growth using the cellophane-based method. Dashed lines and arrows illustrated tendril-like structures.
(TIF)

S2 Fig PA2133 overexpression did not restore TM in the Δ*amrZ* mutant. The empty vector pJN105 or the PA2133-carrying plasmid pJN2133 was introduced in PAO1 or the Δ*amrZ* mutant. TM was measured in the presence of 0.5% arabinose as the inducer using the subsurface twitching method. Scale bar: 5 mm.
(TIF)

S1 Table Strains, plasmids, and oligonucleotides used in this study. (DOCX)

S2 Table Complete gene list from microarray analyses. (XLS)
