# Supplementary figures and images for: Development of a Novel Method for Analyzing Pseudomonas aeruginosa Twitching Motility and Its Application to Define the AmrZ Regulon
Source: PLoS One. 2015 Aug 26;10(8):e0136426. doi: 10.1371/journal.pone.0136426 (PMC4550253; doi:10.1371/journal.pone.0136426)

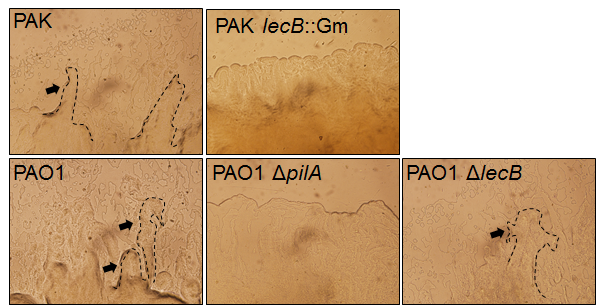

Supplement: S1 Fig — Phase contrast microscopy was used to examine edge subpopulations of PAO1, PAK, and their respective lecB mutants after growth using the cellophane-based method. Dashed lines and arrows illustrated tendril-like structures. (TIF) [file pone.0136426.s001.tif]

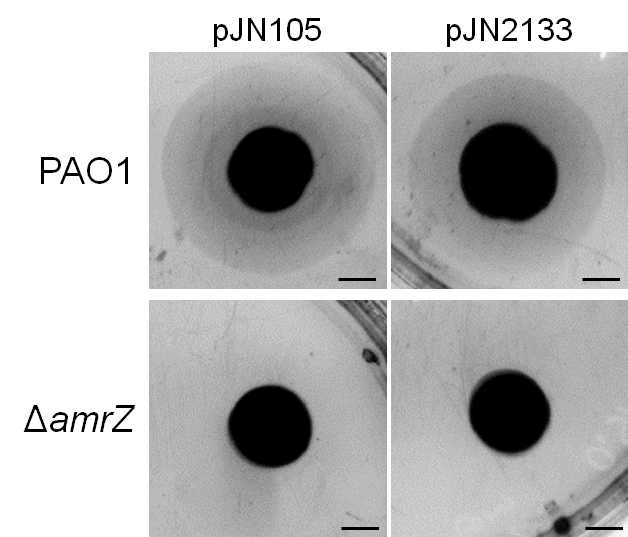

Supplement: S2 Fig — The empty vector pJN105 or the PA2133-carrying plasmid pJN2133 was introduced in PAO1 or the ΔamrZ mutant. TM was measured in the presence of 0.5% arabinose as the inducer using the subsurface twitching method. Scale bar: 5 mm. (TIF) [file pone.0136426.s002.tif]
