# Supplementary material for: Development of a Novel Method for Analyzing Pseudomonas aeruginosa Twitching Motility and Its Application to Define the AmrZ Regulon
Source: PLoS One. 2015 Aug 26;10(8):e0136426. doi: 10.1371/journal.pone.0136426 (PMC4550253; doi:10.1371/journal.pone.0136426)
Supplement: S1 Table — (DOCX) [file pone.0136426.s003.docx]

**Table S1. Strains, plasmids, and oligonucleotides used in this study**

| **Strains/Plasmids** | **Relevant genotype/description/sequence** | **Sources** |
| --- | --- | --- |
| **Strains** |  |  |
| ***Escherichia coli*** |  |  |
|  |  |  |
| NEB5α | fhuA2 Δ(argF-lacZ)U169 phoA glnV44 Φ80 Δ(lacZ)M15 gyrA96 recA1 relA1 endA1 thi-1 hsdR17 | New England Biolabs |
| S17λ*pir* | *pro* *thi* *hsdR*^+^ Tp^R^ Sm^R^; chromosome::RP4-2 Tc::Mu-Kan::Tn7/λ*pir* | (33) |
|  |  |  |
| ***P. aeruginosa*** |  |  |
| PAO1 strain background |  |  |
| PAO1 | *­*PAO1 wild type | (25) |
| WFPA205 | Δ*amrZ* | (25) |
| WFPA513 | AmrZV20A | (25) |
| WFPA510 | *amrZ* complemented in WFPA205 | (25) |
| JJH283 | Δ*fliC* | J.J. Harrison |
| PAO-JP3 | Δ*lasR*&Δ*rhlR* | (62) |
| OSUPA239 | Δ*lecB* | This study |
| OSUPA268 | Δ*pilA* | This study |
| PAK strain background |  |  |
| PAK | PAK wild type | (26) |
| PAK Δ*lecB* | PAK *lecB*::Gm^R^ | (26) |
|  |  |  |
| **Plasmids** |  |  |
| pEX18Ap | Deletion construct backbone. Cb300 | (32) |
| pBX27 | PAO1 *lecB* deletion construct in pEX18Ap. This plasmid was constructed by *lecB*_ABCD primers | This study |
| pBX38 | PAO1 *pilA* deletion construct in pEX18Ap. This plasmid was constructed by *pilA*_ABCD primers | This study |
| pBX39 | PAO1 *amrZ* deletion construct in pEX18Ap. This plasmid was constructed by primers *amrZ*122-125 | This study |
| pHERD20T | Arabinose-inducible vector | (34) |
| p*lecB* (pBX14) | pHERD20T-*lecB*. Primers *lecB_*CDS_F and *lecB_*CDS_R were used to amplify the *lecB* coding sequence | This study |
| pJN105 | Empty vector. Cb300 | (61) |
| pJN2133 | pJN105-PA2133 | (61) |
|  |  |  |
| **Oligonucleotides** |  |  |
|  |  |  |
| *lecB*_CDS_F | cgctctagaAGGCAGGCCAGGTATTCAGTG | This study |
| *lecB*_CDS_R | cgcgcatgcCCATCCCGTCCCTTCCGAAC | This study |
| *pilA*_RT_F | CGACGATCAACCCGCTGAAGAC | This study |
| *pilA*_RT_R | ACTTGTTGGCATCCGGCTCGA | This study |
| *lecA*_RT_F | CGTTTTGTGGTGCGCTGGTC | This study |
| *lecA*_RT_R | TAGGTTCCGGGCACGTCGTT | This study |
| *lecB*_RT_F | AGTGTTCACCCTTCCCGCCA | This study |
| *lecB*_RT_R | GTGCTTTGCCCGCTGAAGGT | This study |
| *pys2*_RT_F | GGAAGCAGAAGCCCAGCGAG | This study |
| *pys2*_RT_R | TGGCGTTGGATTGGTCAGTTGAG | This study |
| *prtN*_RT_F | GAACGCTGGTTTCGCAACCTG | This study |
| *prtN*_RT_R | GGCGCTTCCGGCTTGGTTC | This study |
| *rhlA*_RT_F | CGCTCAACGATCGGGGCTAC | This study |
| *rhlA*_RT_R | CTCCACCCGCGAGAAACTGC | This study |
| *nirS*_RT_F | GCGAATCGTGGAAGGTGCTG | This study |
| *nirS*_RT_R | TGACCTTGACGATCTTCTTGCTG | This study |
| *norC*_RT_F | CCTGACCTACCACACCGAGAAG | This study |
| *norC*_RT_R | GCAGCCGACGCAGTTGTTC | This study |
| *hemH*_RT_F | CGAGAACAGCCGCAACGTCA | This study |
| *hemH*_RT_R | GCGACTGGAACGACACCGAC | This study |
| *hasAp*_RT_F | GACAGCCAGGAGGTGAGCTTC | This study |
| *hasAp*_RT_R | CGTCGATCTGCCCCTGCAG | This study |
| *rpoD*_RT_F | GCCGAGCTGTTCATGCCGAT | (22) |
| *rpoD*_RT_R | GAACAGGCGCAGGAAGTCGG | (22) |
| *lecB*_A | cccctctagaTTGATCGGGAGTCATAGGGTAC | This study |
| *lecB*_B | GTCCCTTCCGAACTCCTAGCGTTGCCATGGTGTATCTCCACT | This study |
| *lecB*_C | AGTGGAGATACACCATGGCAACGCTAGGAGTTCGGAAGGGAC | This study |
| *lecB*_D | ccgaagcttGGAGGTGGCGGTGATCAAC | This study |
| *pilA*_A | tgctctagaGCTTTTCGCTGATGGCGTC | This study |
| *pilA*_B | AACAAGCCACCTTCGATCACCGAATCTCTCCGTTGATTATGTA | This study |
| *pilA*_C | TACATAATCAACGGAGAGATTCGGTGATCGAAGGTGGCTTGTT | This study |
| *pilA*_D | aactgcagCGCATAGCACCCGGCAAG | This study |
| *amrZ*122 | tgctctagaGGAGCGTGGATTTGCCGGAG | This study |
| *amrZ*123 | TACGCGTGGGCTTCGGCGCACATTGAACCTGTAGAGTCAGG | This study |
| *amrZ*124 | CCTGACTCTACAGGTTCAATGTGCGCCGAAGCCCACGCGTA | This study |
| *amrZ*125 | cccaagcttCCACAGATACAGGTAGGCGATC | This study |
| *lecB*_EMSA_F | [6FAM]CGGCATCGGCAATAGATCGTTAC | This study |
| *lecB*_EMSA_R | GAACCGGGTGTTGGCGGGAAG | This study |
| *algB*68 | [6FAM]CGCATGCCAGCCTTTCTGA | This study |
| *algB*69 | TTCACTGCCATTCGGCTTCTG | This study |
| *algD*71 | [6FAM]ACCGTTCGTCTGCAAGTCATG | This study |
| *algD*73 | ACCAACTTGATGGCCTTTCCG | This study |
